# Global Distribution of *Polaromonas* Phylotypes - Evidence for a Highly Successful Dispersal Capacity

**DOI:** 10.1371/journal.pone.0023742

**Published:** 2011-08-29

**Authors:** John L. Darcy, Ryan C. Lynch, Andrew J. King, Michael S. Robeson, Steven K. Schmidt

**Affiliations:** Department of Ecology and Evolutionary Biology, University of Colorado, Boulder, Colorado, United States of America; Université Paris-Sud, France

## Abstract

Bacteria from the genus *Polaromonas* are dominant phylotypes in clone libraries and culture collections from polar and high-elevation environments. Although *Polaromonas* has been found on six continents, we do not know if the same phylotypes exist in all locations or if they exhibit genetic isolation by distance patterns. To examine their biogeographic distribution, we analyzed all available, long-read 16S rRNA gene sequences of *Polaromonas* phylotypes from glacial and periglacial environments across the globe. Using genetic isolation by geographic distance analyses, including Mantel tests and Mantel correlograms, we found that *Polaromonas* phylotypes are globally distributed showing weak isolation by distance patterns at global scales. More focused analyses using discrete, equally sampled distances classes, revealed that only two distance classes (out of 12 total) showed significant spatial structuring. Overall, our analyses show that most *Polaromonas* phylotypes are truly globally distributed, but that some, as yet unknown, environmental variable may be selecting for unique phylotypes at a minority of our global sites. Analyses of aerobiological and genomic data suggest that *Polaromonas* phylotypes are globally distributed as dormant cells through high-elevation air currents; *Polaromonas* phylotypes are common in air and snow samples from high altitudes, and a glacial-ice metagenome and the two sequenced *Polaromonas* genomes contain the gene *hipA*, suggesting that *Polaromonas* can form dormant cells.

## Introduction

Over two decades worth of sequence collection from culture-independent studies of microbial communities has finally provided the necessary data to address questions regarding microbial biogeographic patterns. Hypotheses about whether and why microorganisms have biogeographic patterns, given their small size and ease of dispersal, can now be tested [1], [2]. Most recent studies of microbial biogeography have focused on patterns of genetic markers [3], phylogenetic community structure [4], [5] or broad taxonomic groups [6]. However, sufficient long-read sequence data is now available to gain a better understanding of the global distribution of specific microbial clades [7], even for clades that are not currently cultured [8]. Such an understanding is a first step in describing the ecology and environmental importance of microbes that are presumed to be ubiquitous in similar environments across the globe.

Isolated “extreme” ecosystems, such as glacial and periglacial environments, are ideal for testing biogeographic hypotheses because they contain relatively low microbial diversity and they are geographically widespread. This is especially true of high-elevation environments because, like widespread thermal environments [9], they are often separated by large expanses of temperate habitats, yet occur on every continent [8], [10]. However, unlike thermal environments, cold, high-elevation environments are linked through the upper atmosphere via the movement of cold air masses. Surprisingly few studies have examined the geographic distribution of microbial families, genera, or species in the cryosphere [7], [11], [12], [13] and only one study has focused on the biogeography of microorganisms in extreme high-elevation ecosystems [8].

There is a growing body of evidence that members of the genus *Polaromonas* are among the dominant bacteria of glacial ice and sediments worldwide. They have been isolated from glacial ice [14], sea ice [13], sub-glacial sediments [15], and detected in 16S rRNA gene clone libraries [16]–[18] and metagenomic libraries [19] of glaciers. In addition, Nemergut et al. [10] found *Polaromonas* phylotypes in clone libraries of very recently deglaciated soils but not in soils from the same chronosequence that had been deglaciated for more than four years. Finally, our recent research in the high Himalayas [8], Andes [20], [21], Rocky Mountains (this study) and Alaska Range (this study) suggests that sequences closely aligned with *Polaromonas* are almost ubiquitous in unvegetated periglacial soils in widely separated mountain ranges. Given our recent studies of high elevation periglacial soils and numerous sequences from other studies of glacial sediments worldwide [16]–[19], [22], we determined the biogeographic distribution of *Polaromonas* phylotypes in periglacial sediments and ice across the globe.

## Results

**Figure 1:**
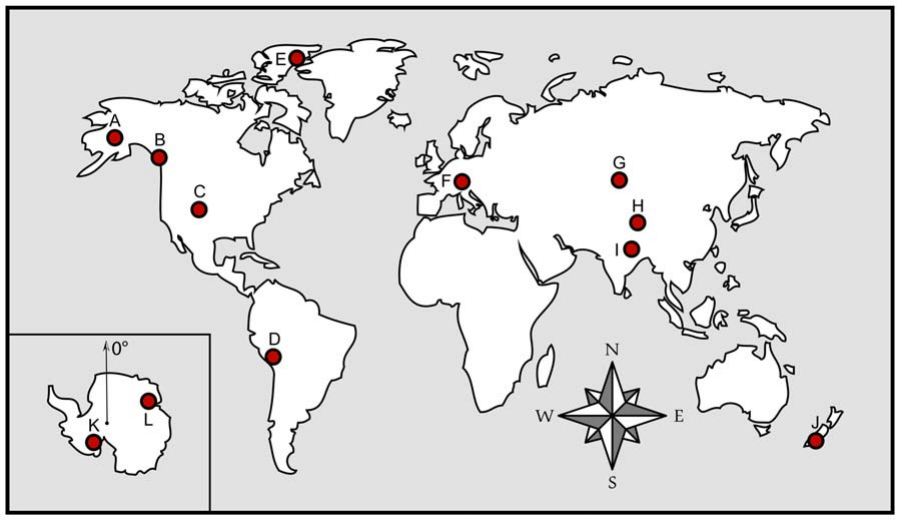
Locations of worldwide sampling sites on 6 continents. Antarctic locations are inset, with Prime Meridian noted. Alphabetic labels (A – L) for location markers correspond to the those of Table 1 and Table S1.

**Figure 2:**
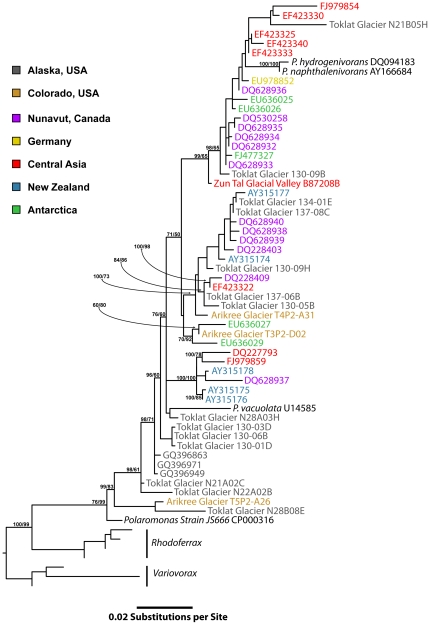
Maximum-likelihood phylogenetic tree showing the diversity, geographic distribution and sub-clade groupings of the *Polaromonas* monophyletic clade. Tree is rooted with sequences of *Rhodoferrax* and *Variovorax*. Nodes with annotations are well supported by both Baysian and maximum-likelihood trees (posterior probability values above 60%, bootstrap support above 60%, respectively). Sequence names or accession numbers are color-coded by geographic regions. Guide sequences of well-described *Polaromonas* species and strains are shown to confirm that the clade depicted is equivalent to the genus *Polaromonas*.

*Polaromonas* sequences from sediments and ice of glaciers worldwide (Figure 1) formed a monophyletic clade that was significantly differentiated from its two closest sister clades, *Rhodoferax* and *Variovorax* [23]. This conclusion was supported both by Bayesian (0.99 posterior probability) and Maximum Likelihood (100% of bootstraps) approaches (Figure 2). Guide sequences from four well-described strains of *Polaromonas* [23]–[26] were encompassed by this clade supporting our use of the term *Polaromonas* for the whole clade. Phylotypes from the two most intensively sampled sites (Toklat Glacier, Alaska and Nunavut, High Arctic) are distributed throughout this clade (Figure 2). For example, phylotypes from Toklat Glacier occurred in both sub-clades of *Polaromonas* and basal to those sub-clades (Figure 2), indicating that *Polaromonas* phylotypes from the Toklat Glacier are also globally distributed.

**Figure 3:**
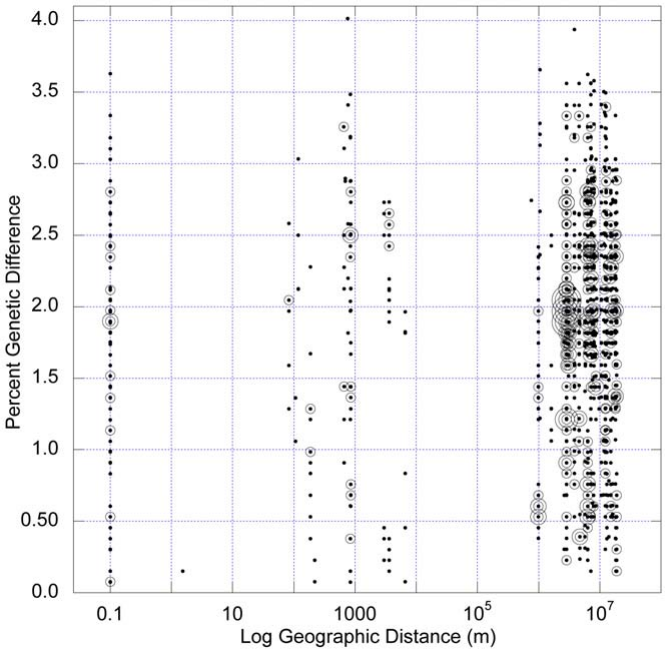
All pairwise comparisons of genetic distance by geographic distance for glacier-associated *Polaromonas* sequences (n = 1378). There was a slight (*r*_M_ = 0.09) but significant (P = 0.01, Mantel test) increase in genetic distance with geographic distance for the entire data set, but a Mantel correlogram (Figure 4) revealed that spatial structuring was not evident in 10 out of 12 total distance classes across the globe. This pattern was maintained even when only the hyper-variable regions of the 16S rRNA gene were analyzed (see Figure S1). The largest geographic distance comparison in this study was 18,838 km, between the Collins Glacier in Antarctica and the John Evans glacier in Nunavut, Canada. Circle size is proportional to the number of pair wise comparisons at each point on the plot, with bin sizes of 1, 2, 3–4, 5, and >5 for the smallest to largest circles.

**Figure 4:**
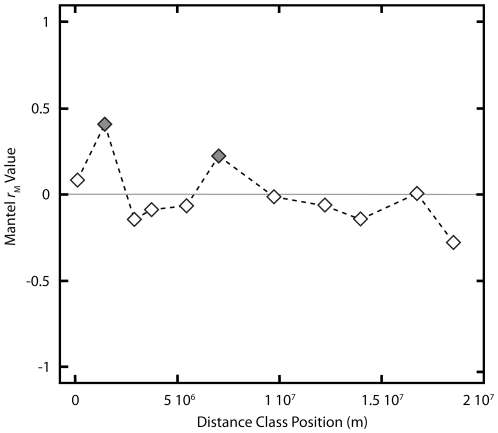
Mantel Correlogram showing spatial structuring of *Polaromonas* phylotypes within different distance classes (the midpoint of each distance class is plotted). Shaded diamonds represent distance classes which contained statistically significant spatial structuring as determined using Mantel test on each distance class, and comparing the test's *P*-value after applying the Bonferroni correction (corrected alpha = 0.004). Unlike previous studies that used this approach to show spatial structuring at large scales [58], our data clearly show that a minority of distance classes indicate significant isolation by distance patterns supporting our contention that *Polaromonas* phylotypes are globally distributed.

To test whether *Polaromonas* phylotypes are globally distributed we examined genetic divergence by geographic distance patterns [8], [27], [28] for pair-wise comparisons among all of the phylotypes in the *Polaromonas* clade from Figure 2. These analyses revealed a high level of genetic overlap among phylotypes (Figure 3) even at the largest geographic distances represented in our analyses (18,830 km; John Evans Glacier in the Arctic to Collins Glacier in Antarctica) confirming that at least some phylotypes of *Polaromonas* are globally distributed to glacial environments. A Mantel correlogram [29] (Figure 4) revealed that significant (*P*<0.004), Bonferroni-corrected) patterns of genetic divergence occurred within only two distinct distance classes, meaning that within the bounds of these classes genetic distance is positively correlated with geographic distance for *Polaromonas* phylotypes. The first significantly correlated distance class spans pairwise distances of 6.5 kilometers to 2882 kilometers, and the second spans pairwise distances of 6471 kilometers to 7588 kilometers. All five larger distance classes (>7588 km) showed no significant isolation by distance patterns, indicating that some, as yet unknown, environmental factor may drive genetic differences at the two shorter distances classes mentioned above. However, the fact that 10 out of the 12 distance classes resulted in non-significant Mantel tests is strong evidence for the global dispersal of *Polaromonas* phylotypes.

Comparison among the genomes of *P. naphthalenivorans*, *Polaromonas* sp. strain JS666, and the metagenome from the Northern Schneeferner Glacier [19] allowed us to infer what genes may contribute to global dispersal. *Polaromonas* was one of dominant contributors to annotated proteins and 16S phylotypes from the Northern Schneeferner Glacier metagenome; out of the 508,960 classifiable protein-coding genes, 48,922, or 9.3% were aligned to the *Polaromonas* sp. strain JS666 genome with a maximum allowable E-value of 1e^−05^. This provides coverage of 3,820 of the total 5,656 genomic features (68%). In addition to the expected presence of genes that help *Polaromonas* cope with the osmotic and oxidative stress of glacial life, the occurrence of at least one dormancy inducing gene, *hip*A, was established in all three genomic datasets. Since all *Polaromonas* genomes lack the necessary genes for spore formation, we suggest they use an alternative dormancy mechanism, which also provides a high capacity for successful airborne dispersal.

## Discussion

The goal of this study was to statistically describe the global biogeographic distribution of known *Polaromonas* phylotypes in glacial environments in order to better understand their role in the cryosphere and to determine if they are endemic to each site or are globally dispersed. We restricted our analyses to long sequence lengths (>1280 BP) that were in a monophyletic clade (Figure 2) bound at basal and distal levels by described *Polaromonas* spp. [23]–[26]. These and other precautions (see methods) insure that the biogeographic patterns we observed are not due to inclusion of misidentified *Polaromonas* sequences that would artificially inflate genetic distance by geographic distance comparisons. This focused phylogenetic approach also allows us to shed new light on whether *Polaromonas* phylotypes are globally dispersed. Are they being constantly distributed globally or are they endemic to individual sites?

Overall our results show that some *Polaromonas* phylotypes are globally distributed to glacial environments, but that some unmeasured environmental variable may be influencing spatial structuring of *Polaromonas* phylotypes within a minority (2 out of 12) of distance classes tested. Testing the influence of environmental variables is not possible at the present time because we do not have access to raw samples from most of the sites, but future research could elucidate what factors, in addition to geographic distance, contribute to the genetic structuring of *Polaromonas* phylotypes. Most importantly however, the present study shows that *Polaromonas* phylotypes are more widely dispersed than other microbial clades that have been studied using a phylogenetic approach similar to that employed here. For example, recent studies of Bacteria and Archaea show stronger patterns of apparent endemicity [8], [9], [28] than we observed, as do microbial eukaryotes in glacial [8] and temperate environments [5]. Indeed, Robeson et al. [5] found spatial autocorrelation ranges of about a hundred meters for soil rotifer phylotypes indicating that they are many orders of magnitude less globally dispersed than the *Polaromonas* clade studied here.

Another strong indicator of the global distribution of *Polaromonas* phylotypes comes from comparing the within site variability to the global variability of phylotypes. For example, the most intensively sampled site in our analyses was the Toklat Glacier in Alaska, and Figure 2 shows that phylotypes from this site are spread throughout the *Polaromonas* phylogeny. In addition, different sequences from the Toklat Glacier are almost identical to those from glaciers in the Arctic [17], [22], Tibet (unpubl.) and New Zealand [15] among others (Figure 2). In other words, the local diversity of *Polaromonas* phylotypes is roughly equivalent to the global diversity with both spatial scales showing a 4% total range in genetic distances.

The apparent global distribution of *Polaromonas* phylotypes shown here raises the question of how these Gram-negative bacteria with no known spores are dispersed and if there is a global locus from which they are originating. An interesting consideration is the presence of the *hip*A gene in the two publically-available *Polaromonas* genomes, as well as in the only metagenome of glacial ice [19]. *HipA* is linked to the formation metabolically dormant cells [30] as it phosphorylates elongation-factor Tu (EF-TU), thereby blocking translation and inducing dormancy [31]. Recent cell sorting efforts for *E. coli* persister cells demonstrated highly reduced translation and apparent smaller cell sizes compared to vegetative cells that had high levels of translation [32]. Interestingly, in all species tested in culture so far, formation of persister cells follows a similar pattern to sporulation: low occurrence during mid-log phase followed by a significant increase in persister cell formation during stationary phase [32], [33]. Whether or not the formation of persister cells via *hipA* in *Polaromonas* provides an easily dispersible propagule or whether it is found in all *Polaromonas* phylotypes remain open but testable hypotheses.

At present there is not enough information to indicate that *Polaromonas* phylotypes originate from any one locus or habitat type, but the genus has been found in many non-glacial soils and sediments and are often characterized as being psychrotrophic rather than psychrophilic [23], [24], [26], [34], [35]. It is therefore possible that glacial *Polaromonas* species are being transported from temperate soils and sediments and are not indigenous to glacial and periglacial environments where they could just be persisting as dormant cells. More physiologic and genomic [19] studies of glacial *Polaromonas* species are needed to determine if specific glacial phylotypes exist.

The unexpected global distribution pattern of *Polaromonas* phylotypes in high-elevation periglacial environments also suggests that they are being transported there in the upper atmosphere. Although independent confirmation of this is sparse, Fahlgren et al. [36] found *Polaromonas* sequences in air samples using two different sampling devises on Fløyen Mountain, Norway but not in samples taken at sea level. In addition, *Polaromonas* is a common inhabitant of snow sampled at very high elevations. Hervàs and Casamayor [37] found *Polaromonas* sequences ( = clade GKS16 [38]) deposited in surface snow in the highest reaches of the Pyrenees Mountains and Liu et al. [39] found that *Polaromonas* was only one of two genera found in all snow clone libraries from 4 geographically distributed sites on the Tibetan Plateau. The hypothesis that *Polaromonas* is being dispersed in high altitude air currents and snow also explains why Liu et al. [14] found *Polaromonas* of decreasing importance in clone libraries in the order of supra-glacial snow>glacial ice>glacial melt water. Obviously more work is needed to understand the aerobiology of *Polaromonas* species, but our biogeographic analyses and the studies discussed above are consistent with the hypothesis that *Polaromonas* species are transported across great distances in the atmosphere.

Finally, recent studies of non-glacial (but seasonally cold) environments shed some light on the possible roles of *Polaromonas* in glacial systems. Strains of *Polaromonas* from seasonally cold soils are able to oxidize a wide array of unusual energy sources including H_2_ [26], arsenite [34] and a broad range of recalcitrant organic compounds [24], [25], [35]. Furthermore, genomic studies are revealing that this extreme metabolic versatility may be due to high levels of horizontal gene transfer [35] allowing *Polaromonas* to adapt to shifting availabilities of energy sources in periglacial environments. Thus, a picture is emerging of *Polaromonas* as a metabolically diverse “opportunitroph” [40], [41] that takes advantage of transient periods of higher temperatures and substrate availability that occur in all but the most extreme glacial environments.

Taken together, our biogeographic analyses, aerobiological studies, and genomic data allow us to deduce a probable explanation for the unusual distribution of *Polaromonas* phylotypes in glacial systems. Members of this clade are globally dispersed and show as much genetic diversity within an environment as they do across the globe. This high level of local diversity combined with their apparent propensity for rapid evolution through horizontal gene transfer [35] allows them to adapt to shifting environmental gradients (freeze-thaw cycles, extreme drying and physical disruption due to glacial movement) common in high alpine environments [21], [42]. These shifting environmental gradients likely result in periodic decimation of local populations, allowing for the establishment of *Polaromonas* phylotypes from the atmosphere. This continuous input of widely dispersed phylotypes would result in the weak genetic isolation by distance patterns observed in the present study. However, the ecological role of *Polaromonas* spp. in high altitude sediments, ice and the atmosphere remains an unsolved mystery.

## Methods

To describe the global biogeographic distribution of *Polaromonas* phylotypes we used previously published and unpublished sequences (see Table 1 for references) from GenBank [43] and new sequences that we obtained from periglacial sediments from our previously described sites in the Himalayas [44], Colorado Rocky Mountains [45], Andes [21] and Alaska [4]. Sediment samples (0 to 4 cm deep) were collected sterilely during the summer at all sites in a grid pattern in order to obtain spatial representation as described in King et al. [4], [46]. The location of each sample site was logged using a Garmin 60CSx gps unit. Samples were frozen in the field and shipped to Colorado where they were kept at −80°C until DNA was extracted. Figure 1 shows the global sites used in these analyses.

**Table 1:** Locations, accession numbers, and publication sources for 16S rRNA gene sequences.

Region	Site	Geographic Coordinates	Accession #s	Publication Source	On Fig. 1
Alaska, USA	Toklat glacier	63.39 N 149.91 W	JF719324-28, 31–38, JF729309	This study	A
	Mendenhall glacier	58.435837 N 134.5546 W	GQ396863, 949, 971	Sattin et al. [58]	B
Colorado, USA	Arikaree glacier	40.057276 N 105.6432 W	JF719322, 3, 9	This study	C
Peru	Cordillera Vilcanota	13.770123 S 71.07002 W	GQ306091	Schmidt et al. [21]	D
Nunavut, Canada	John Evans glacier	79.66 N 74 W	DQ228409, 3	Skidmore et al. [17]	E
	John Evans glacier	79.63 N 74.38 W	DQ628932-40, DQ530258	Cheng et al. [22]	E
Germany	Schneeferner glacier	47.42 N 10.98 E	EU978852	Simon et al. [19]	F
Tain-Shen, China	Glacier no. 1	43.15 N 86.87 E	EF423322, 25, 30, 33, 40	Wang et al. (unpubl.)	G
	Glacier no. 1	43.15 N 86.87 E	FJ979854, 9	Zhang et al. (unpubl.)	G
Nepal	Zuntal glacial valley	28.7210 N 83.9155 E	JF719330	This study	H
Tibet, China	Puruogangri ice field	33.89 N 89.15 E	DQ227793	Zhang et al [18]	I
New Zealand	Fox glacier	45.51 S 170.14 E	AY315176, 7	Foght et al. [15]	J
	Franz Josef glacier	43.48 S 170.21 E	AY315174, 5, 8	Foght et al. [15]	J
Antarctica	Kamb ice stream	82.25 S 145 W	FJ477327	Lanoil et al. [16]	K
	Collins glacier	73.21 S 66.97 E	EU636024-9	Garcia et al. (unpubl.)	L

Labels in the rightmost column correspond to Figure 1, where sample sites are shown to be globally distributed.

To extract DNA, 0.4 grams of sediment from each sample was processed with the Mo Bio PowerSoil™ DNA isolation Kit (Carlsbad, CA, USA) and 3 µl of each extraction was PCR amplified in 25 µl reaction volumes using primers 8F (5′-agagtttgatcctggctcag-3′) and 1391R (5′-gacgggcggtgwgtrca-3′) [8]. The reaction conditions consisted of 1 µM of each primer, 250 µM each dA, dT, dG, dC, 0.25 µL bovine serum albumen, 1 unit of OmniKlen™ Taq polymerase, and 3 µl DNA extract as template. For negative controls, sterile Millipore water was used as template. Denaturing temperature was 94°C (1 minute), the annealing temperature was 53°C (30 seconds) and the extension temperature was 72°C (2 minutes 30 seconds). PCR products were purified using the Quiaquick gel extraction protocol (Qiagen, Valencia, CA, USA), with HyperLadder II™ as a reference. Plasmids were cloned into OneShot™ *E. coli* using the Invitrogen Topo TA™ cloning kit (Invitrogen, Carlsbad, CA, USA). Colonies were grown on selective media for 18 hours, pelleted and sent overnight on dry ice to Functional Biosciences (Madison, WI, USA) and sequenced bi-directionally using sequencing primers T7 and M13R.

Sequencher 4.6 (Gene Codes Co., Ann Arbor, MI, USA) was used to interpret the chromatograms, edit out unreliable data, and assemble contigs. Sequences were imported into ARB v. 9.4 [47], and aligned using the SILVA reference database [48]. Sequences unique to this study were deposited in GenBank [43] under accession numbers JF719322-JF719338 and JF729309. Other glacial *Polaromonas* sequences, as well as known *Polaromonas* guide sequences were downloaded from GenBank and imported into ARB. All *Polaromonas* sequences were aligned in ARB, and then filtered by base frequency to exclude any position in the alignment that had below 30% identity across all sequences. Sequences were exported from ARB to a FASTA file, and Mesquite [49] was used to convert between file formats.

Phylogenetic trees were constructed using two robust methods in order to clearly define a well-supported *Polaromonas* clade. RAxML [50] was used to make a maximum likelihood (ML) trees with 500 bootstraps, and MrBayes [51]–[53] was run for 5 million generations at a temperature value of 0.02. The Bayes and ML trees were then compared for structural similarity and mutual support of the node separating the out-group genera (*Variovorax* and *Rhodoferax*) from *Polaromonas* phylotypes. The Bayes tree had a posterior probability of 0.99 for this node, and in the ML tree, 100% of bootstraps contained that node, confirming that the *Polaromonas* clade discussed below is indeed monophyletic.

Genomic and metagenomic comparisons were performed using the RAST and MG-RAST annotation and comparison platforms, which utilize the manually curated SEED database [54], [55]. The genomes of *Polaromonas naphthalenivorans CJ2 (*CP000529) [35] and *Polaromonas* sp. JS666 (CP000316) [25], as well as the glacier metagenome (SRX000607) [19] were obtained from the NCBI database. Comparisons of annotated metabolic subsystems between the genomic data were sorted by identity and function.

Geographic distances between sample sites were computed in R [56] using the Fields package [57] and used to construct a geographic distance matrix using in-house software. An uncorrected genetic distance matrix was exported from ARB using the same filter that was used to export sequences for the trees. To test for a correlation between these matrices, Mantel tests were performed in R using 1000 randomized permutations per test. A Mantel correlogram was constructed to the specifications set forth by Legendre and Legendre [29], [59]. The application of Sturge's rule resulted in the data being partitioned into 12 distances classes each containing 115 pairwise comparisons so that each distance class had the same statistical power. Mantel tests were carried out on each of the 12 distance classes, and a Bonferroni correction was applied to the original alpha value of 0.05 resulting in a corrected alpha value of 0.004. Using this approach, only 2 of the 12 distances classes showed significant *P*-values (*P*<0.004), however the first distance class was not testable (showed a null result for P and *r*_M_ values) because it contained no geographic variation. Guide sequences and the outgroup sequences were not included in biogeographic analyses. Several groupings of our *Polaromonas* sequences were tested in this manner, to see whether variables in addition to geographic distance, such as sequence length, distance from glaciers, elevation, or whether the sequences were obtained from culture-dependent or culture-independent studies were correlated with spatial structuring. However at the present time we do not have enough environmental data to disentangle the effects of geographic distance from environmental variation across the 2 distance classes that showed significant spatial structuring.

## Supporting Information

Figure S1 **All pairwise comparisons of genetic distance (only hyper-variable regions of the 16S rRNA gene) by geographic distance for glacier-associated** ***Polaromonas*** **sequences (n = 1378).** There was a significant (P = 0.016, Mantel test) increase in genetic distance with geographic distance for the entire data. Circle size is proportional to the number of pair wise comparisons at each point on the plot, with bin sizes of 1, 2, 3–4, 5, and >5 for the smallest to largest circles.
(TIF)

Table S1 **Distances in kilometers between sites on** **Figure 1** **and** **Table 1** **.**
(DOC)

## References

[1] Bell T, Ager D, Song J-I, Newman JA, Thompson IP (2005). Larger islands house more bacterial taxa.. Science.

[2] Martiny JBH, Bohannan BJM, Brown JH, Colwell RK, Fuhrman JA (2006). Microbial biogeography: putting microorganisms on the map.. Nat Rev Microbiol.

[3] Fierer N, Jackson RB (2006). The diversity and biogeography of soil bacterial communities.. Proc Natl Acad Sci USA.

[4] King AJ, Freeman KR, McCormick KF, Lynch RC, Lozupone C (2010). Biogeography and habitat modelling of high-alpine bacteria.. Nat Commun.

[5] Robeson MS, King AJ, Freeman KR, Birky CW, Martin AP (2011). Soil rotifer communities are extremely diverse globally but spatially autocorrelated locally.. Proc Natl Acad Sci USA.

[6] Nemergut DR, Costello EK, Hamady M, Lozupone C, Jiang L (2011). Global patterns in the biogeography of bacterial taxa.. Environ Microbiol.

[7] Rodrigues DF, da C Jesus E, Ayala-del-Rio HL, Pellizari VH, Gilichinsky D (2009). Biogeography of two cold-adapted genera: *Psychrobacter* and *Exiguobacterium*.. ISME J.

[8] Schmidt SK, Lynch RC, King AJ, Karki D, Robeson MS (2011). Phylogeography of microbial phototrophs in the dry valleys of the high Himalayas and Antarctica.. Proc Roy Soc B.

[9] Papke RT, Ramsing NB, Bateson MM, Ward DM (2003). Geographical isolation in hot spring cyanobacteria.. Environ Microbiol.

[10] Nemergut DR, Anderson SP, Cleveland CC, Martin AP, Miller AE (2007). Microbial community succession in an unvegetated, recently deglaciated soil.. Microb Ecol.

[11] Bowman JP, McCuaig RD (2003). Biodiversity, community structural shifts, and biogeography of prokaryotes within Antarctic continental shelf sediment.. Appl Environ Microbiol.

[12] Brinkmeyer R, Knittel K, Jürgens J, Weyland H, Amann R, Helmke E (2003). Diversity and structure of bacterial communities in Arctic versus Antarctic pack ice.. Appl Environ Microbiol.

[13] Staley JT, Gosink JJ (1999). Poles apart: Biodiversity and biogeography of sea ice bacteria.. Ann Rev Microbiol.

[14] Liu Y, Yao T, Kang S, Jiao N, Zeng Y (2007). Microbial community structure in major habitats above 6000 m on Mount Everest.. Chinese Sci Bull.

[15] Foght J, Aislabie J, Turner S, Brown CE, Ryburn J (2004). Culturable bacteria in subglacial sediments and ice from two Southern Hemisphere glaciers.. Microb Ecol.

[16] Lanoil B, Skidmore M, Priscu JC, Han S, Foo W (2009). Bacteria beneath the West Antarctic Ice Sheet.. Environ Microbiol.

[17] Skidmore M, Anderson SP, Sharp M, Foght J, Lanoil BD (2005). Comparison of microbial community compositions of two subglacial environments reveals a possible role for microbes in chemical weathering processes.. Appl Environ Microbiol.

[18] Zhang X, Yao T, Tian L, Xu S, An L (2008). Phylogenetic and physiological diversity of bacteria isolated from Puruogangri ice core.. Microb Ecol.

[19] Simon C, Wiezer A, Strittmatter AW, Daniel R (2009). Phylogenetic diversity and metabolic potential revealed in a glacier ice metagenome.. Appl Environ Microbiol.

[20] Schmidt SK, Reed SC, Nemergut DR, Grandy AS, Cleveland CC (2008). The earliest stages of ecosystem succession in high-elevation (5000 metres above sea level), recently deglaciated soils.. Proc Roy Soc B.

[21] Schmidt SK, Nemergut DR, Miller AE, Freeman KR, King AJ (2009). Microbial activity and diversity during extreme freeze–thaw cycles in periglacial soils, 5400 m elevation, Cordillera Vilcanota, Perú.. Extremophiles.

[22] Cheng SM, Foght JM (2007). Cultivation-independent and -dependent characterization of bacteria resident beneath John Evans Glacier.. FEMS Microbiol Ecol.

[23] Irgens RL, Gosink JJ, Staley JT (1996). *Polaromonas vacuolata* gen. nov., sp. nov., a psychrophilic, marine, gas vacuolate bacterium from Antarctica.. Int J Syst Bacteriol.

[24] Jeon CO, Park W, Padmanabhan P, DeRito C, Snape JR (2003). Discovery of a bacterium, with distinctive dioxygenase, that is responsible for in situ biodegradation in contaminated sediment.. Proc Natl Acad Sci USA.

[25] Mattes TE, Alexander AK, Richardson PM, Munk AC, Han CS (2008). The Genome of *Polaromonas* sp. Strain JS666: Insights into the evolution of a hydrocarbon- and xenobiotic-degrading bacterium, and features of relevance to biotechnology.. Appl Environ Microbiol.

[26] Sizova M, Panikov N (2007). *Polaromonas hydrogenivorans* sp. nov., a psychrotolerant hydrogen-oxidizing bacterium from Alaskan soil.. Int J Syst Evol Microbiol.

[27] Oline DK, Schmidt SK, Grant MC (2006). Biogeography and landscape-scale diversity of the dominant Crenarchaeota of soil.. Microb Ecol.

[28] Whitaker RJ, Grogan DW, Taylor JW (2003). Geographic barriers isolate endemic populations of hyperthermophilic Archaea.. Science.

[29] Legendre P, Legendre L (1998). Numerical Ecology, Second English Edition.

[30] Harrison JJ, Ceri H, Roper NJ, Badry EA, Sproule KM (2005). Persister cells mediate tolerance to metal oxyanions in *Escherichia coli*.. Microbiol.

[31] Black DS, Irwin B, Moyed HS (1994). Autoregulation of *hip*, an operon that affects lethality due to inhibition of peptidoglycan or DNA synthesis.. J Bacteriol.

[32] Lewis K (2010). Persister Cells.. Ann Rev Microbiol.

[33] Spoering AL, Lewis K (2001). Biofilms and planktonic cells of *Pseudomonas aeruginosa* have similar resistance to killing by antimicrobials.. J Bacteriol.

[34] Osborne T, Jamieson H, Hudson-Edwards K, Nordstrom DK, Walker S (2010). Microbial oxidation of arsenite in a subarctic environment: diversity of arsenite oxidase genes and identification of a psychrotolerant arsenite oxidiser.. BMC Microbiology.

[35] Yagi JM, Sims D, Brettin T, Bruce D, Madsen EL (2009). The genome of *Polaromonas naphthalenivorans* strain CJ2, isolated from coal tar-contaminated sediment, reveals physiological and metabolic versatility and evolution through extensive horizontal gene transfer.. Environ Microbiol.

[36] Fahlgren C, Bratbak G, Sandaa R-A, Thyrhaug R, Zweifel U (2010). Diversity of airborne bacteria in samples collected using different devices for aerosol collection.. Aerobiologia.

[37] Hervàs A, Casamayor EO (2009). High similarity between bacterioneuston and airborne bacterial community compositions in a high mountain lake area.. FEMS Microbiol Ecol.

[38] Zwart G, Crump BC, Kamst-van Agterveld MP, Hagen F, Han SK (2002). Typical freshwater bacteria: an analysis of available 16S rRNA gene sequences from plankton of lakes and rivers.. Aquat Microb Ecol.

[39] Liu Y, Yao T, Jiao N, Kang S, Xu B (2009). Bacterial diversity in the snow over Tibetan Plateau glaciers.. Extremophiles.

[40] Moran MA, Buchan A, Gonzalez JM, Heidelberg JF, Whitman WB (2004). Genome sequence of *Silicibacter pomeroyi* reveals adaptations to the marine environment.. Nature.

[41] Polz MF, Hunt DE, Preheim SP, Weinreich DM (2006). Patterns and mechanisms of genetic and phenotypic differentiation in marine microbes.. Phil Trans R Soc B.

[42] Meyer AF, Lipson DA, Martin AP, Schadt CW, Schmidt SK (2004). Molecular and metabolic characterization of cold-tolerant alpine soil *Pseudomonas* sensu stricto.. Appl Environ Microbiol.

[43] Benson DA, Karsch-Mizrachi I, Lipman DJ, Ostell J, Rapp BA (2000). GenBank.. Nucleic Acids Res.

[44] Freeman KR, Martin AP, Karki D, Lynch RC, Mitter MS (2009). Evidence that chytrids dominate fungal communities in high-elevation soils.. Proc Natl Acad Sci USA.

[45] Ley RE, Williams MW, Schmidt SK (2004). Microbial population dynamics in an extreme environment: controlling factors in talus soils at 3750 m in the Colorado Rocky Mountains.. Biogeochem.

[46] King AJ, Meyer AF, Schmidt SK (2008). High levels of microbial biomass and activity in unvegetated tropical and temperate alpine soils.. Soil Biol Biochem.

[47] Ludwig W, Strunk O, Westram R, Richter L, Meier H (2004). ARB: a software environment for sequence data.. Nucleic Acids Res.

[48] Pruesse E, Quast C, Knittel K, Fuchs BM, Ludwig W (2007). SILVA: a comprehensive online resource for quality checked and aligned ribosomal RNA sequence data compatible with ARB.. Nucleic Acids Res.

[49] Maddison W, Maddison DR (2001).

[50] Stamatakis A, Ludwig T, Meier H (2005). RAxML-III: a fast program for maximum likelihood-based inference of large phylogenetic trees.. Bioinformatics.

[51] Ronquist F, Huelsenbeck JP (2003). MrBayes 3: Bayesian phylogenetic inference under mixed models.. Bioinformatics.

[52] Huelsenbeck JP, Ronquist F, Nielsen R, Bollback JP (2001). Bayesian inference of phylogeny and its impact on evolutionary biology.. Science.

[53] Altekar G, Dwarkadas S, Huelsenbeck JP, Ronquist F (2004). Parallel metropolis coupled Markov chain Monte Carlo for Bayesian phylogenetic inference.. Bioinformatics.

[54] Aziz R, Bartels D, Best A, DeJongh M, Disz T (2008). The RAST Server: Rapid annotations using subsystems technology.. BMC Genomics.

[55] Meyer F, Paarmann D, D'Souza M, Olson R, Glass EM (2008). The metagenomics RAST server - a public resource for the automatic phylogenetic and functional analysis of metagenomes.. BMC Bioinformatics.

[56] R Core Development Team (2008).

[57] Furrer R, Nuchka D, Sain S (2007).

[58] Sattin S, Cleveland CC, Hood E, Reed SC, King AJ (2009). Functional shifts in unvegetated, perhumid, recently-deglaciated soils do not correlate with shifts in soil bacterial community composition.. J Microbiol.

[59] Cho J, Tiedje J (2000). Biogeography and degree of endemicity of fluorescent *Pseudomonas* strains in soil.. Appl Environ Microbiol.

