# Supplementary figures and images for: Global Distribution of Polaromonas Phylotypes - Evidence for a Highly Successful Dispersal Capacity
Source: PLoS One. 2011 Aug 29;6(8):e23742. doi: 10.1371/journal.pone.0023742 (PMC3163589; doi:10.1371/journal.pone.0023742)

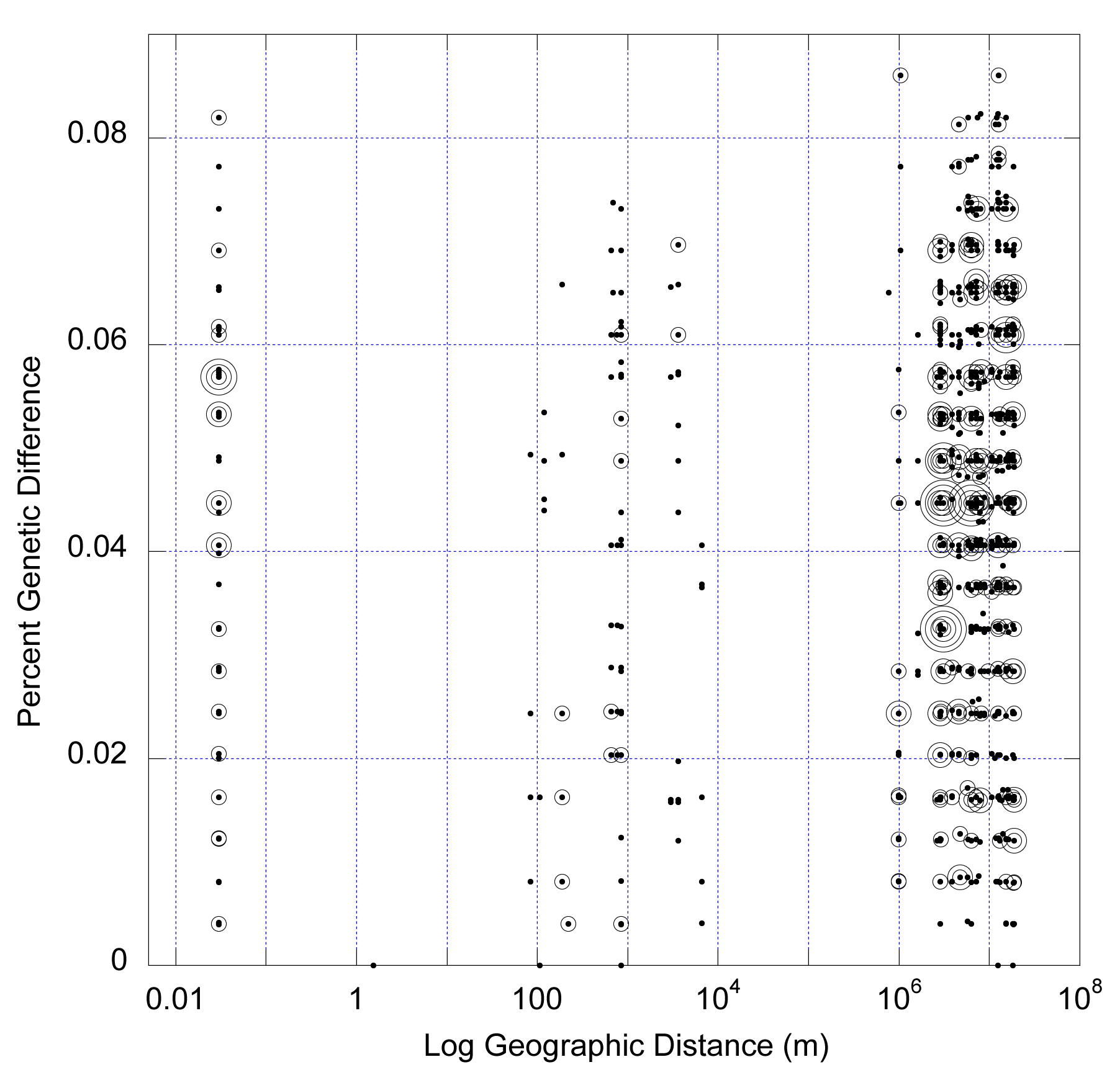

Supplement: Figure S1 — All pairwise comparisons of genetic distance (only hyper-variable regions of the 16S rRNA gene) by geographic distance for glacier-associated Polaromonas sequences (n = 1378). There was a significant (P = 0.016, Mantel test) increase in genetic distance with geographic distance for the entire data. Circle size is proportional to the number of pair wise comparisons at each point on the plot, with bin sizes of 1, 2, 3–4, 5, and >5 for the smallest to largest circles. (TIF) [file pone.0023742.s001.tif]
