# Supplementary material for: Global Distribution of Polaromonas Phylotypes - Evidence for a Highly Successful Dispersal Capacity
Source: PLoS One. 2011 Aug 29;6(8):e23742. doi: 10.1371/journal.pone.0023742 (PMC3163589; doi:10.1371/journal.pone.0023742)
Supplement: Table S1 — Distances in kilometers between sites on Figure 1 and Table 1 . (DOC) [file pone.0023742.s002.doc]

**Supplementary Table 1. Distances in kilometers between sites on Figure 1 and Table 1.**

|  | A | B | C | D | E | F | G | H | I | J | K | L |
| --- | --- | --- | --- | --- | --- | --- | --- | --- | --- | --- | --- | --- |
| A | 0 | 993 | 3877 | 10842 | 2890 | 7588 | 7167 | 8749 | 8026 | 12624 | 16216 | 18216 |
| B | 993 | 0 | 2891 | 9865 | 3097 | 7838 | 8100 | 9700 | 8988 | 12637 | 15673 | 18148 |
| C | 3877 | 2891 | 0 | 6978 | 4609 | 8461 | 10690 | 12318 | 11667 | 12679 | 13794 | 16326 |
| D | 10842 | 9865 | 6978 | 0 | 10402 | 10558 | 16146 | 16966 | 17038 | 11031 | 8261 | 9896 |
| E | 2890 | 3097 | 4609 | 10402 | 0 | 4753 | 6312 | 7895 | 7353 | 15493 | 18392 | 18830 |
| F | 7588 | 7838 | 8461 | 10558 | 4753 | 0 | 5722 | 6471 | 6488 | 18431 | 16074 | 14089 |
| G | 7167 | 8100 | 10690 | 16146 | 6312 | 5722 | 0 | 1628 | 1050 | 12840 | 15318 | 13044 |
| H | 8749 | 9700 | 12318 | 16966 | 7895 | 6471 | 1628 | 0 | 761 | 11978 | 13763 | 11419 |
| I | 8026 | 8988 | 11667 | 17038 | 7353 | 6488 | 1050 | 761 | 0 | 12007 | 14269 | 12042 |
| J | 12624 | 12637 | 12679 | 11031 | 15493 | 18431 | 12840 | 11978 | 12007 | 0 | 4375 | 5616 |
| K | 16216 | 15673 | 13794 | 8261 | 18392 | 16074 | 15318 | 13763 | 14269 | 4375 | 0 | 2640 |
| L | 18216 | 18148 | 16326 | 9896 | 18830 | 14089 | 13044 | 11419 | 12042 | 5616 | 2640 | 0 |
